# Predicting full-scale and verbal intelligence scores from functional Connectomic data in individuals with autism Spectrum disorder

**DOI:** 10.1007/s11682-019-00111-w

**Published:** 2019-05-04

**Authors:** Elizabeth Dryburgh, Stephen McKenna, Islem Rekik

**Affiliations:** 1 BASIRA Lab, CVIP Group, Computing, School of Science and Engineering, University of Dundee, Dundee, UK; 2 CVIP Group, Computing, School of Science and Engineering, University of Dundee, Dundee, UK; 3 Faculty of Computer and Informatics, Istanbul Technical University, Istanbul, Turkey

**Keywords:** Autism spectrum disorder, Functional connectivity, Feature selection, Resting-state fMRI, Connectome-based prediction modelling, Intelligence scores

## Abstract

Decoding how intelligence is engrained in the human brain construct is vital in the understanding of particular neurological disorders. While the majority of existing studies focus on characterizing intelligence in neurotypical (NT) brains, investigating how neural correlates of intelligence scores are altered by atypical neurodevelopmental disorders, such as Autism Spectrum Disorders (ASD), is almost absent. To help fill this gap, we use a connectome-based predictive model (CPM) to predict intelligence scores from functional connectome data, derived from resting-state functional magnetic resonance imaging (rsfMRI). The utilized model learns how to select the most significant positive and negative brain connections, independently, to predict the target intelligence scores in NT and ASD populations, respectively. In the first step, using leave-one-out cross-validation we train a linear regressor robust to outliers to identify functional brain connections that best predict the target intelligence score (*p − value <* 0*.*01). Next, for each training subject, positive (respectively negative) connections are summed to produce single-subject positive (respectively negative) summary values. These are then paired with the target training scores to train two linear regressors: (a) a positive model which maps each *positive* summary value to the subject score, and (b) a negative model which maps each negative summary value to the target score. In the testing stage, by selecting the same connections for the left-out testing subject, we compute their positive and negative summary values, which are then fed to the trained negative and positive models for predicting the target score. This framework was applied to NT and ASD populations independently to identify significant functional connections coding for full-scale and verbal intelligence quotients in the brain.

## Introduction

Autism is a spectrum neurodevelopmental disorder associated with social interaction difficulties and repetitive behaviors. Recently, Autism Spectrum Disorder (ASD) cases have increased to 1/160 children globally, according to the World Health Organization (WHO). The centers for disease control and prevention (CDC) estimate nearly 1 in 59 children in the US have ASD. A massive limitation of ASD diagnosis is the breadth of its symptoms as well as a lack of understanding of how it affects the brain-behavior relationship (Worley and Matson 2012). Intelligence, in particular, is an intriguing aspect of ASD, that has not been investigated in-depth from a brain connectomic perspective, with the exception of a recent study using resting-state functional MRI (rs-fMRI) to examine different brain networks underlying intelligence in ASD and NT children (Pua et al. 2017; Wu et al. 2013). Although this work is pioneering, identifying ASD brains that correlated with fluid intelligence, it was not based on predictive brain-behavior modeling. State-of-the-art methods investigating brain-behavior relationship generally used very simple techniques such as correlation or regression model to identify the most relevant brain features coding for the target behavior in a hypothesis-driven manner (Shen et al. 2017). Such methods usually focus on a single connection, region or network of interest and are not data-driven (Whelan and Garavan 2014), which limits the predictive power of the model by ignoring significant connections. Furthermore, taking the absolute values of functional brain connections disregards relevant negative correlations between regions of interest (ROIs) (Shen et al. 2017; Finn et al. 2015). To address these issues, a *cross-validated predictive model* that is data-driven can be utilized to efficiently identify brain connections related to the target behavior. From the standpoint of scientific rigor, cross-validation is designed to infer the presence of a brain behavior relationship more conservatively than correlation, and protects against overfitting by testing the generalizability of the discovered brain features coding for the target relationship using hidden testing samples (Shen et al. 2017).

In this study, we specifically adopt the recently developed connectome-based predictive model (CPM) (Shen et al. 2017), using a functional connectome-driven approach. We build cross-validated models that take functional brain connectomes and outputs predictions of two target intelligence scores: full-scale intelligence (FIQ) and verbal intelligence quotient (VIQ). As highlighted in (Shen et al. 2017), CPM has two appealing aspects in comparison to advanced machine learning techniques: (1) from a practical standpoint, CPM is simpler to implement and requires less expertise in machine learning, which makes it more accessible to the general neuroimaging and neuroscience community for performing cross-validated and replicable data-driven analyses of the brain behavior relationships; and (2) compared with multivariate methods and advanced feature selection and dimensionality reduction techniques for machine learning model training, CPM provides clearly interpretable one-to-one mapping back to the original feature space so that the underlying brain connections contributing to the model can be easily visualized and investigated. This nicely addresses the limitations of feature projection techniques such as non-linear principal component analysis (Shams and Rahman 2011) or feature graph-based embedding techniques (Morris and Rekik 2017). Although such approaches might better capture non-linear relationships between brain features and behavioral scores, they do not facilitate tracking connectomic features to know which connections in the brain are altered by a particular disorder. Therefore, there is no physical meaning for the mapped features as they are intractable although they contribute to boosting the target prediction task.

The modelling procedure comprises of the following steps: (i) connectional feature selection, (ii) feature summarization, (iii) model building, and (iv) model evaluation. CPM learns how to select the most significant positive and negative brain connections, independently, to predict the target FIQ and VIQ intelligence scores in NT and ASD populations, respectively. In the first step, using leave-one-out cross-validation, we train a linear regressor robust to outliers to identify functional brain connections that best predict the target intelligence score. In fact, CPM identifies significant connections by applying a threshold (*p* = 0*.*01). These connections must be present across all leave-one-out cross-validation runs. Next, for each training subject, positive (respectively negative) connections are summed to produce single-subject positive (respectively negative) summary values. These are then paired with the target training scores to train two linear regressors: (a) a positive model which maps each positive summary value to the subject score, and (b) a negative model which maps each negative summary value to the target score. Both models are evaluated using line of best fit and the prediction of all models are scored using r-squared (*r*^2^). In the testing stage, by selecting the same connections for the left-out testing subject, we compute their positive and negative summary values, which are then fed to the trained negative and positive models for predicting the target score. This framework was applied to NT and ASD populations independently to identify significant functional connections coding for full-scale and verbal intelligence quotient in the brain. Specifically, the top five brain connections are identified for both models that best predict target intelligence scores in NT and ASD populations, respectively.

## Materials and methods

### Materials and data preprocessing

We used multi-site rs-fMRI data from the Autism Brain Imaging Data Exchange (ABIDE) I Preprocessed (Craddock et al. 2013). Data from ABIDE has been preprocessed by five different teams using: the Connectome Computation System (CSS), the Configurable Pipeline for the Analysis of Connectomes (CPAC), the Data Processing Assistant for Resting-State fMRI (DPARSF) and the Neuroimaging Analysis Kit. Preprocessed rs-fMRI ABIDE data-sets are subject to quality assessment protocols (based on measures) such as standardized root-mean-squared change in fMRI signal between volumes (DVARs), mean deviation (Mean FD), entropy focus criterion (EFC), full-width half maximum (FWHM) and functional outliers. ABIDE pre-processed data-sets are available online.^1^

Subjects were all male with no significant differences between age (Table 1). FIQ and VIQ exhibited significant differences between NT and ASD groups. Means and standard deviations (SDs) were computed after excluding any missing values in individual subject phenotypes. ASD subjects had been diagnosed based on the autism criteria sets in the Diagnostic and Statistical Manual of Mental Disorders, 4th Edition (DSM-IV-TR) (Zwaigenbaum et al. 2015). Each brain image was parcellated into 116 regions of interest (ROIs) using automatic labelling atlas (AAL) template (Tzourio-Mazoyer et al. 2002). Then, functional connectomes, represented by 116 *×* 116 symmetric matrices, were generated for each subject. The weight (or strength) of a connection between two ROIs *i* and *j* represents Pearson correlation between the average rs-fMRI signal measured in ROI *i* and ROI *j*, respectively.

**Table 1:** Demographic Information for ASD and NT Subjects

Subjects	Age (years)	FIQ	VIQ
ASD (*N* = 202)	15.0 *±* 3.6	106.5 *±* 15.2	105.7 *±* 15.2
NT (*N* = 226)	15.4 *±* 3.8	111.7 *±* 12.0	112.7 *±* 12.0
*p*	0.35	1.9 *×* 10^*−*7^	2.6 *×* 10^*−*6^

*ASD* autism spectrum disorder, *NT* neurotypical, *FIQ* full-scale intelligence quotient, *VIQ* verbal intelligence quotient; *p*: statistical level was calculated using two-tailed two-sample t-test

### Methods

**Fig. 1:**
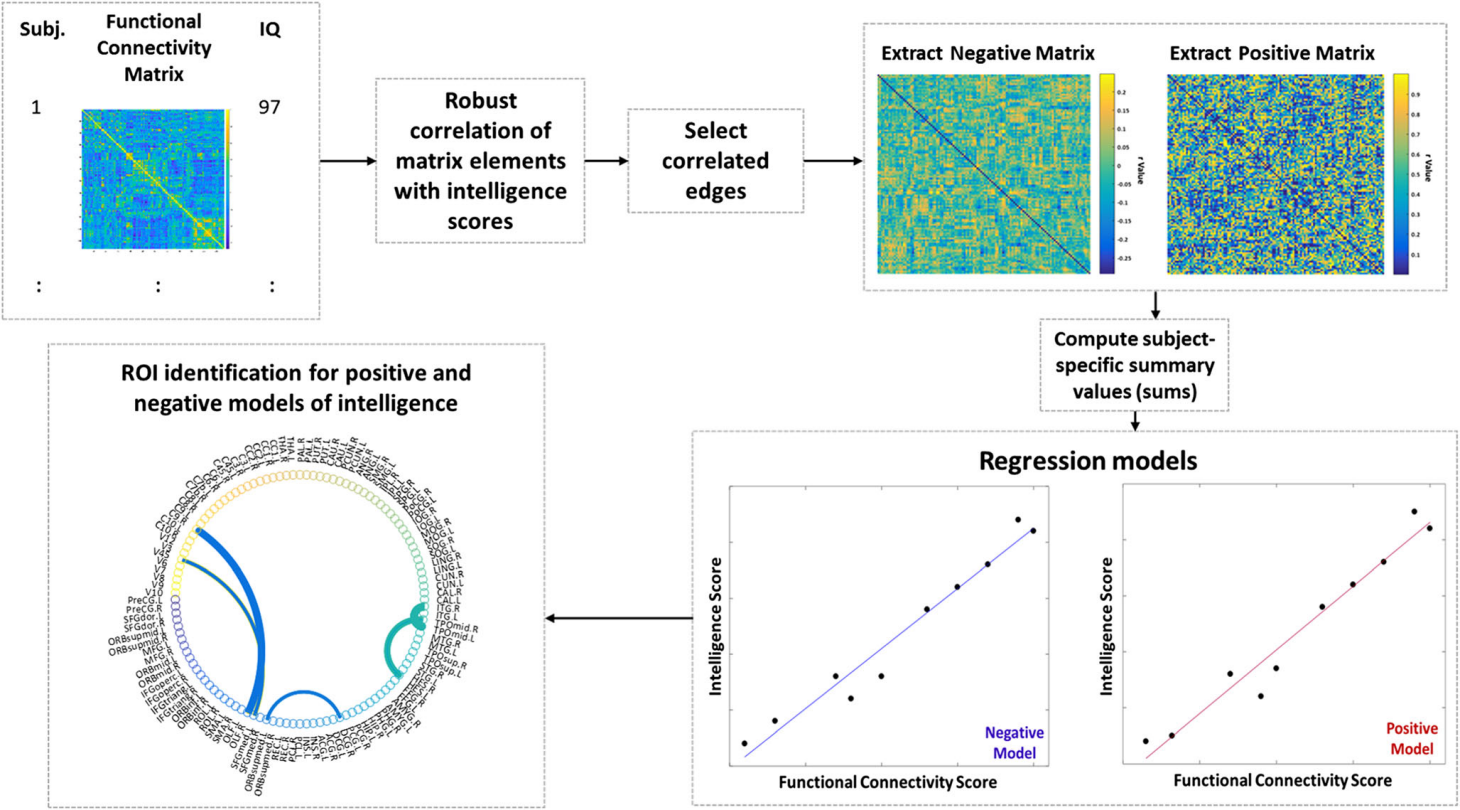
Framework for intelligence score prediction from functional connectomes*.* The Connectome-based Prediction Model (CPM) (Shen et al. 2017) uses 116 by 116 connectivity matrices with corresponding intelligence scores to first train a robust linear regressor in a leave-one-out cross-validation fashion. A brain-behavior relationship is learned by correlating functional brain connections with intelligence scores in the training stage. We select correlated connections with *p*-values below a predefined threshold (*p <* 0*.*01). Selected connections for each training subject are then split into two separate sets: (i) significant positive correlations stored in a positive connectivity matrix and (ii) significant negative (inverse) correlations stored in a negative connectivity matrix. For each training subject, we sum the connection strengths and generate a positive and negative subject-specific summary values, respectively. Ultimately, we train pairs of regressions models: (i) a positive regression model mapping positive summary values to the target intelligence score, and (ii) a negative regression model mapping negative summary values to the target intelligence score. In the testing stage, we test both learned models on the left-out subject to predict the intelligence score of interest. These regression models also identify brain connections that consistently correlate with intelligence scores (bottom left circular graph)

For our brain-intelligence prediction task, we followed the CPM protocol introduced in (Shen et al. 2017). Figure 1 illustrates CPM key steps.

Step 1: *Extracting positive and negative brain connections that correlate with target intelligence scores.* Our input data were 116 by 116 connectivity matrices per subject per cohort. First, we split the functional connectome data into training and testing sets. Given a population of *N* subjects, we use leave-one-out cross-validation strategy, where (*N −* 1) subjects are used for training and one subject is left out for testing. ASD and NT experiments are performed separately to spot most significant brain connections correlated with intelligence scores, independently. We use robust regression, reducing the presence of outliers, to compute the correlation coefficient *r* of each feature (i.e., the weight of an edge connecting two brain regions) with the target intelligence score and derive its statistical significance *p − value* from the training samples. After each functional brain connection is correlated with an intelligence score per subject, significant connections are identified by applying a threshold of *p* = 0*.*01. We extract for each training subject positively and negatively correlated connections, each stored in a matrix (i.e., positive and negative matrices). This produces a negative matrix which will be used to build the negative predictive model and a positive matrix for positive model building (Fig. 1). This allows to tell apart positive and negative brain connections that code for the target intelligence score. In the testing stage, we also extract both negative and positive matrices for the left-out subject.

- Remark 1: The main idea of teasing apart positive and negative connections in our analyses is to prevent bias when interpreting the connectome data. For example, previous connectome studies (Pua et al. 2017; Wu et al. 2013) have used absolute values which eliminates negative correlations. This in turn disregards the inverse relationship between two ROIs which may be important in the understanding of brain connectivity. In this work, we followed the lead of Finn et al. (Finn et al. 2015), which is one of the first studies to address this by creating and assessing both positive and negative correlations separately.

Step 2: *Subject-specific summary value.* The number of selected significant connections varies across subjects; this hinders the training of a linear regressors since they require all training and testing subjects to have the same number of features (connections in this context). To solve this issue, we represent each training individual by two summary values: (i) a positive summary value computed by summing all positive values in the positive subject-specific matrix, and (ii) a negative summary value computed by summing all negative values in the negative subject-specific matrix. Furthermore, as stated in (Shen et al. 2017), one advantage of the single-summary value is that we do not need to use a binary threshold for the separate positive and negative functional brain connection set.
Step 3: *Univariate linear regression.* CPM assumes a linear relationship between the single-subject summary values and behavioral variables. We use the summary values to train a linear regression model, which maps each summary value to the target intelligence score. Hence, we define a positive and negative regression models, which map positive and negative summary values to the target intelligence score, respectively. Models are evaluated by using line of best fit to determine if there is a positive correlation between the predicted IQ scores and actual IQ scores for each cohort. The correlation coefficient r-squared (*r*^2^) is used to evaluate prediction performance of all models.

- Remark 2: We purposefully build a model for each population independently to identify population-specific biomarkers linked with intelligence. Our goal is not to classify ASD and NT subjects nor to identify shared connections coding for intelligence in the brain. For this reason, we do not group them together. Specifically, in this study, we aim to identify the most relevant functional brain connections that code for intelligence scores in healthy and autistic populations, independently. Hence, training is performed on each individual population to discover what biological information the positive (or negative) regression models used. Mixing the data would influence the training of the regressors as they would not be able to isolate population-specific intelligence markers.

Step 4: *Score prediction for unseen data.* For each unseen testing subject, we generate a summary value based on the most significant brain connections identified in the training stage. Next, we input it into the trained regression model to predict its target score. This is applied to positive and negative summary values independently using both learned regression models.
Step 5: *Identifying positive and negative functional brain connections associated with intelligence.* Using the robust linear regressor with a pre-defined *p − value* (Step 1), we identify the most significantly correlated positive (resp. negative) brain connections with the target intelligence score. Since the identified connections might vary across the *N* cross-validation runs, we rank them based on their frequency and correlation values across all runs. Specifically, for a given brain connectivity *f*_*k*_, we calculate its normalized rank across all *N* LOO-CV as follows: $r\left({f}_k\right)=\left({\sum}_{i=1}^N{\sum}_{j=1}^{n_f}{\delta}_{ij}|{r}_{ij}\left({f}_k\right)|\right)/N$, where *δ*_*ij*_ = 1 if *f*_*k*_ is selected out of *n*_*f*_ features, *δ*_*ij*_ = 0 otherwise. The weight ∣*r*_*ij*_(*f*_*k*_)∣ denotes the absolute correlation value of feature *f*_*k*_ with the target behavioral score (Step 1), at the *i*^*th*^ LOO and *j*^*th*^ rank. Next, we identify the top five brain connectivities as features with the top normalized ranks. We note that the ranking process is implemented for negative and positive models independently.

## Results

### Predicted FIQ and VIQ scores

**Fig. 2:**
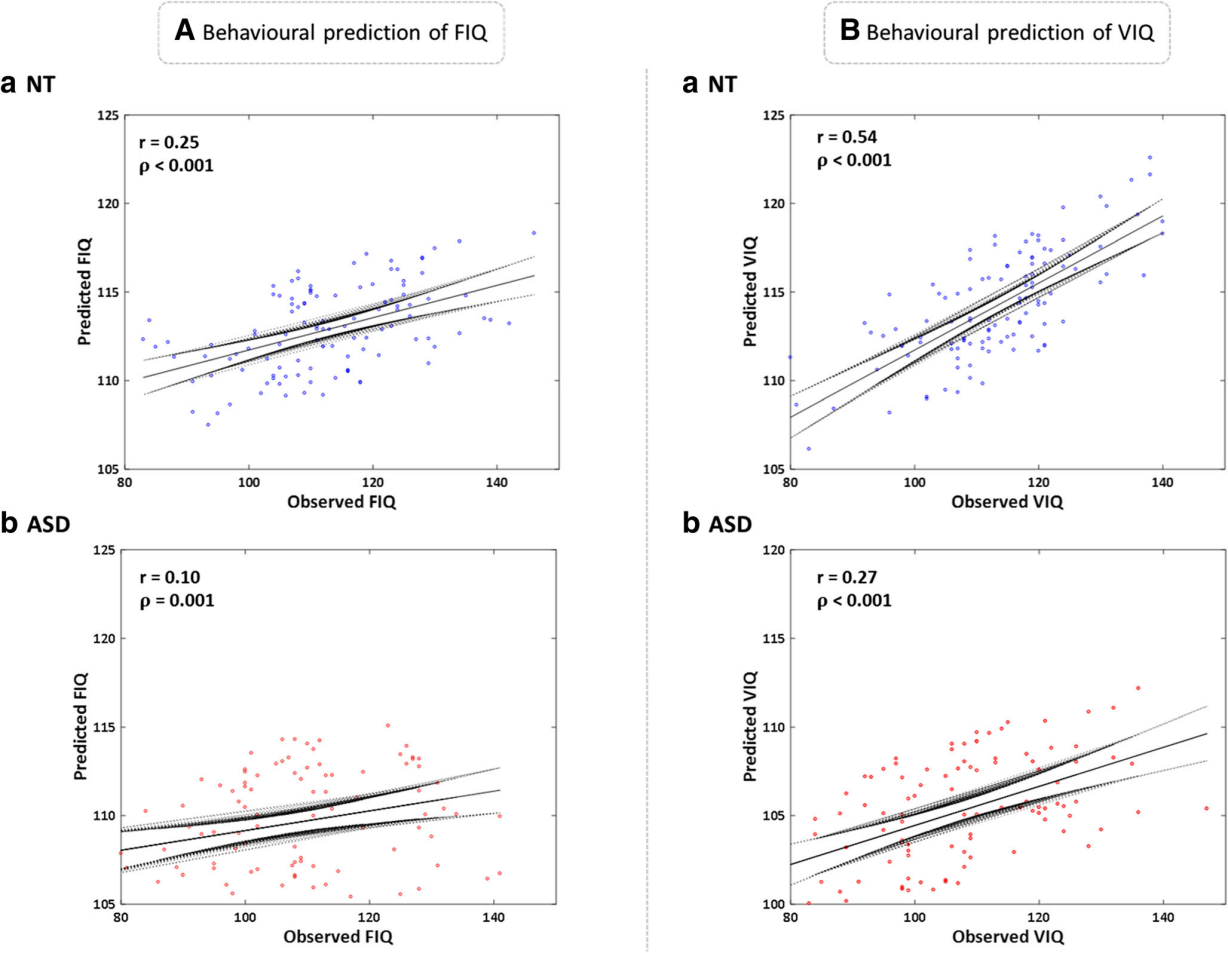
Predicted FIQ and VIQ scores*.* (**a**) *a*) linear correlation plot for observed and predicted NT FIQ (*N* = 202) (*r* = 0*.*25) (*p <* 0*.*001) with 95% confidence intervals. b) ASD FIQ (*N* = 226) (*r* = 0*.*10) (*p <* 0*.*01). (**b**) *a*) linear correlation plot for observed and predicted NT VIQ (*N* = 226) (*r* = 0*.*54) (*p <* 0*.*001) with 95% confidence intervals. *b*) ASD FIQ (*N* = 202) (*r* = 0*.*27) (*p <* 0*.*001)

For each regression model trained using leave-one-out cross-validation, we assessed both the square of the Pearson correlation coefficient or r-squared coefficient *r*^2^ and its associated *p* value. If this prediction probability is lower than the conventional 1% (*p <* 0*.*01), the correlation coefficient is called statistically significant. Both models of intelligence exhibited significant prediction of target intelligence scores. FIQ prediction for ASD (*p <* 0*.*01) was lower (*r* = 0*.*10) than for NT (*p <* 0*.*001) (*r* = 0*.*25). Both models exhibited an increasing relationship between the dependent and independent variables. VIQ models demonstrated stronger linear relationships for ASD (*p <* 0*.*001) (*r* = 0*.*27) and NT (*p <* 0*.*001) (*r* = 0*.*54) (Figure 2).

**Fig. 3:**
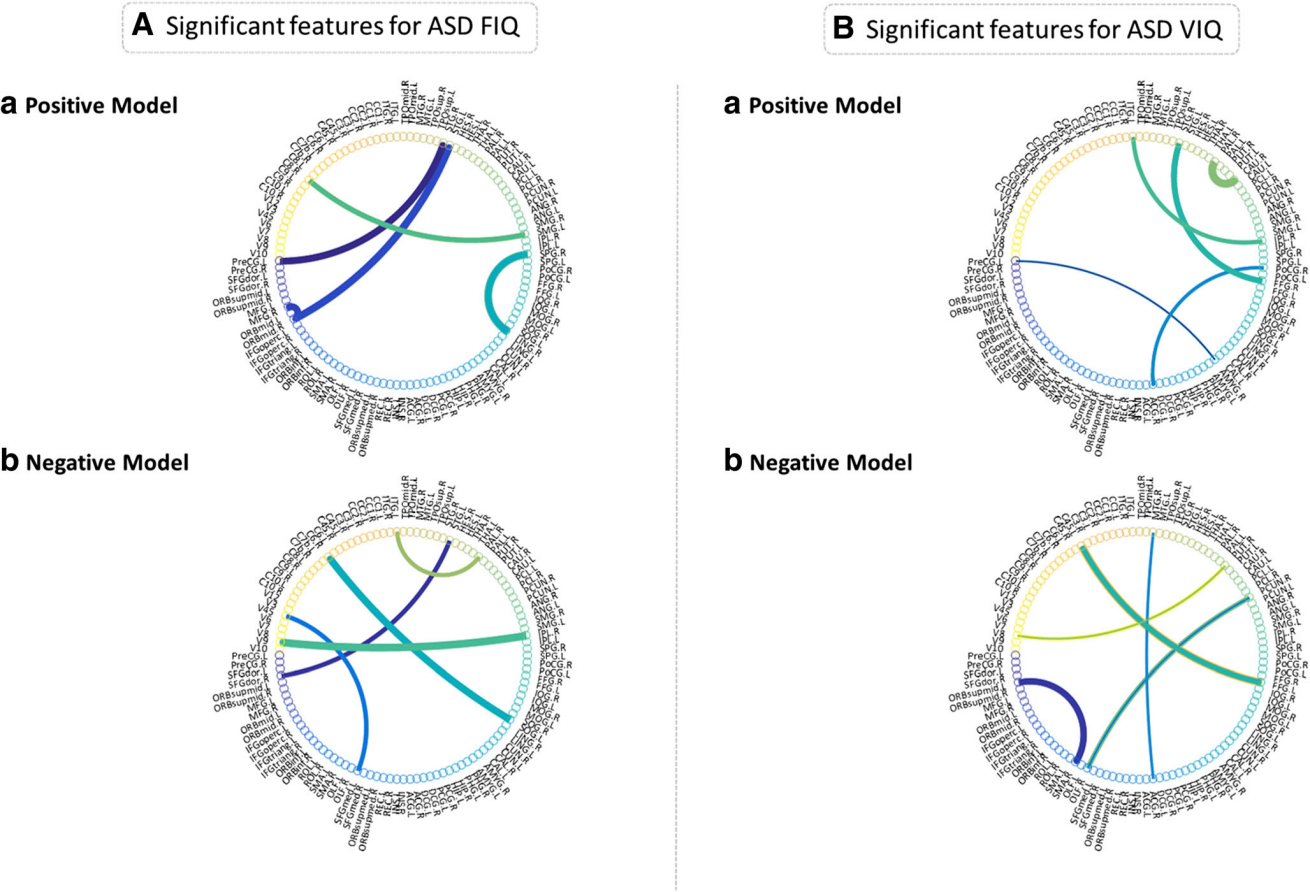
Selected ROIs (*p* < 0.01) for ASD FIQ and VIQ scores*.* (**a**: FIQ) *a*) Positive ROIs. ROIs are ranked by strongest connectivity strength, represented as denser connections between two pairs of ROIs. ROIs for this model were parietal, temporal and frontal connections. Parietal ROIs were: right superior parietal gyrus (SPG.R) and left supramarginal gyrus (SMG.L). Temporal ROIs were: right superior temporal gyrus (STG.R) and STG.L. Frontal ROIs were: right middle frontal gyrus (MFG.R) and right middle frontal gyrus, orbital part (Orbmid.R). *b*) Negative ROIs. ROIs for this model were vermis, frontal and temporal. Vermis ROIs: lobule IX of vermis (V9) and lobule IV, V of vermis (V45). Frontal ROIs: left superior frontal gyrus, medial (SFGmed.L) and right superior frontal gyrus, dorsolateral (SFGdor.R). Temporal ROIs: left superior temporal gyrus (STG.L) and left inferior temporal gyrus (ITG.L). (**b**: VIQ) *a*) Positive ROIs. ROIs for this model were temporal and sensorimotor. Temporal ROIs: right fusiform gyrus (FFG.R), STG.R and left inferior temporal gyrus (ITG.L). Sensorimotor ROIs: right postcentral gyrus (PoCG.R) and left precentral gyrus (PreCG.L). *b*) Negative ROIs. ROIs for this model were temporal and frontal connections. Temporal ROIs: FFG.L and right middle temporal gyrus (MTG.R). Frontal ROIs: left superior frontal gyrus, medial orbital (Orbsupmid.L) and right olfactory cortex (OLF.R)

Tentative ROIs for each model are selected based on connection strength, i.e., graphs. Thick edges represent strong functional connectivity between two ROIs, while thin edges denote weak functional connectivity between ROIs (Figure 3). The top five pairs of ROIs for the positive ASD model were: 1) right lingual gyrus (Ling.R) to right superior parietal gyrus (SPG.R), 2) right middle frontal gyrus (MFG.R) to right middle frontal gyrus, orbital part (Orbmid.R), 3) left precentral gyrus (PreCG.L) to right superior temporal gyrus (STG.R), 4) Orbmid. R to STG.L and 5) left supramarginal gyrus (SMG.L) to left lobule VIII of cerebellar hemisphere (C8.L). Positive ROIs were parietal (1,5), temporal (3,4) and frontal (2,4). The top five functional connections for the negative model involved the: 1) left superior occipital gyrus (SOG.L) to right lobule VI of cerebellar hemisphere (C6.R), 2) right inferior parietal (IPL.R) to lobule IX of vermis (V9), 3) left superior frontal gyrus, medial (SFGmed.L) to lobule IV, V of vermis (V45), 4) right superior frontal gyrus, dorsolateral (SFGdor.R) to STG.L and 5) left lenticular nucleus, pallidum (PAL.L) to left inferior temporal gyrus (ITG.L). The negative model included vermis (2,3), frontal (3,4) and temporal (4,5) ROIs. Frontal and temporal connections are highlighted in both models. However, vermis connections are only apparent in the negative ASD FIQ model.

Similarly, the top five functional brain connections were identified for VIQ ASD models (Figure 3). For the positive model these were: 1) right caudate nucleus (CAU.R) to PAL.R, 2) right fusiform gyrus (FFG.R) to STG.R, 3) IPL.R to left inferior temporal gyrus (ITG.L), 4) right anterior cingulate and paracingulate gyri (ACG.R) to right postcentral gyrus (PoCG.R) and 5) PreCG.L to right amygdala (AMYG.R). ROIs of the positive model were temporal (2,3) and sensorimotor (4,5). The Top five negative functional connections were: 1) FFG.L to C45.L, 2) left superior frontal gyrus, medial orbital (Orbsupmid.L) to right supplementary area (SMA.R), 3) right olfactory cortex (OLF.R) to right precuneus (PCUN.R), 4) ACG.R to right middle temporal gyrus (MTG.R) and 5) right lenticular nucleus, putamen (PUT.R) to lobule VIII of vermis (V8). The main ROIs of the negative model were occipitotemporal (1), temporal (4) and frontal (2,3) connections. Both models exhibit temporal ROIs. However, the positive model involved sensorimotor (4,5) compared to the negative model’s frontal ROIs (2,3).

## Discussion

We investigated how positive and negative neural correlates derived from functional MRI are predictive of intelligence scores in both typical and autistic brains. The utilized brain-behavior prediction model (Shen et al. 2017) suggests distinct hallmarks for ASD and NT populations.

### Model intelligence scores in NT and ASD populations

Our linear models (Fig. 2) suggest predictive correlations for FIQ and VIQ models. Intelligence models for the NT population exhibit higher *r* scores than ASD. Table 1 shows higher variation in IQ scores for ASD than the NT cohort. ASD samples from ABIDE are biased towards acquisition of data from high-functioning individuals (Craddock et al. 2013). These factors may affect CPM intelligence prediction for ASD subjects more than our NT cohort.

### Unravelling functional connections most correlated with intelligence scores

Next, we wanted to identify specific features that contributed to direct correlations observed in Fig. 2. Like other studies, we identified both positive and negative characteristics directly associated with models of FIQ or VIQ (Wu et al. 2013; Finn et al. 2015). As stated previously, using absolute values of a connectome ignores the negative biological correlations.

The positive ASD FIQ model (Fig. 3Aa) highlighted frontal, temporal and parietal ROIs. There is evidence from the literature to support the fronto-parietal (P-FIT model) for relevance to intelligence and other findings from temporal and midbrain regions (Jung and Haier 2007; Luders et al. 2009). Our FIQ models did not include direct fronto-parietal connections but the ASD cohort did include frontal and parietal ROIs. Frontal ROIs of the positive model include: 2) MFG.R (frontal) to Orbmid.R (frontal) and 4) Orbmid.R (frontal) to STG.L (temporal). Similarly, parietal ROIs: 1) Ling.R (occipital) to SPG.R (parietal) and 5) SMG.L (parietal) to C8.L (cerebellum). Interestingly, both ASD and NT subjects exhibited connections for LING; reported to be activated in fMRI tasks associated with the visual processing of words and human faces (Cheng et al. 2015). As stated, ASD feature 1 demonstrates an ROI pairing of the LING.R to SPG.R but in our NT model SMA.L (left supplementary motor area)(frontal) connects to LING.R. Therefore, LING.R is present for both NT and ASD positive FIQ models but with differing pairings. ASD studies have reported higher activation of parietal and occipital regions; suggesting stronger visual orientated intelligence during task-based fMRI (Kana et al. 2006; Simard et al. 2015; Shafai et al. 2015). The visuospatial pathway is coordinated by two cortical routes: recognizing objects (occipital-temporal) and detecting the location of objects (partial-occipital) (Sahyoun et al. 2010). Therefore, abnormal activation of these ROIs may be associated with defects in the visuospatial pathway for ASD in relation to intelligence. However, it is difficult to state based on the selection of the top five pairs of ROIs for each model

Our negative ASD FIQ model (Fig. 3Ab) included vermis, frontal and temporal ROIs. Three of these connections: 1) SOG.L (occipital) to C6.R (cerebellum), 2) IPL.R (parietal) to V9 (vermis), 3) SFGmed.L (frontal) to V45 (vermis) are associated with either the vermis or cerebellum. The vermis is the median portion of cerebellum and connects both cerebellar hemispheres; these are regarded as cerebellar ROIs (Bullmore and Sporns 2009). Similarly, our negative NT model exhibited one cerebellar feature: right caudate nucleus (CAU.R) to left lobule IX of cerebellar hemisphere (C9.L). Pezoulas et. al (2017) assessed 137 NT subjects focusing on a parcelled cerebellum; they reported evidence for small-world properties in high IQ females. Significant small-worldness is associated with high clustering coefficient of neighboring nodes (Bullmore and Sporns 2009). These connections have been postulated to be important for intelligence (Pezoulas et al. 2017). Finn et. al (2015) reported negative cerebellar connections in association to intelligence with FIQ. However, our ASD negative FIQ model exhibited three cerebellar features compared to one for NT. The exact reasons for a higher number cerebellar ROIs in our negative ASD FIQ model is unknown.

The positive VIQ model Fig. 3Ba; included temporal and sensorimoter ROIs. Temporal ROIs were: 2) FFG.R (temporal) to STG.R (temporal), FFG has been implicated in recognition of known faces. This is critical for VIQ; as one engages in conversation it is vital to recognize facial body language of the other participant to then act in the most appropriate manner (Salmond et al. 2005; Pierce et al. 2001). Pierce et. al (2001) assessed haemodynamic responses of the MTG, FFG, amygdala and IOG (inferior temporal gyrus), during a face perception task. From these ROIs; the amygdala only exhibited anatomical differences but the FFG presented unique connectivity patterns. For example, NT subjects demonstrate maximal activity in fusiform face area (FFA) compared to weaker scattered activity in ASD. Pierce et.al concluded that the FFA is activated weakly for ASD subjects. Based on our results, we report altered connectivity for facial recognition areas for ASD.

Interestingly, our NT cohort exhibited 3/5 ROIs as cingulate regions. 1) Left inferior frontal gyrus, triangular part (IFGtriang.L) to right posterior cingulate gyrus (PCG.R), 2) left median cingulate and paracingulate gyri (DCG.L) to right superior parietal gyrus (SPG.R) and 3) right median cingulate and paracingulate gyri (DCG.R) to right amygdala (AMYG.R). Kilroy et. al (2011) reported positive correlations with VIQ and cingulate cortical activity in healthy participants. The cingulate receives inputs from the thalamus and limbic system; involved in emotional processing. Comparatively, our ASD group exhibited one cingulate ROI in the positive model; 4) ACG.R (cingulate) to PoCG.R (sensorimotor). Altered cingulate processing in ASD may contribute to increased emotional outbursts observed in autism (Mazefsky et al. 2013).

Our negative ASD VIQ model exhibited two frontal and temporal ROIs. Frontal connections: 2) Orbsupmid.L (frontal) to SMA.R (sensorimoter) and 3) OLF.R (frontal) to PCUN.R (parietal). Interestingly, precuneus activity has been highlighted in many ASD studies and is associated with the theory of mind (ToM). Mental imagery manifested by the self occurs by using episodic memory, known to be defective in ASD (Cheng et al. 2015; Irimia et al. 2017; Cheng et al. 2017; Zielinski et al. 2012). ASD is hypothesized to be a defect with the process of spontaneous mentalizing. ToM areas are active when representing someone else’s mental states: desires, beliefs and intentions. During the false-belief task NT individuals are able to spontaneously mentalize compared to ASD children (Cheng et al. 2015, 2017; Nijhof et al. 2018). Altered activity in the precuneus may contribute to difficulties associated with using VIQ socially in ASD.

### Unravelling shared functional connections most correlated with intelligence scores across ASD and NT populations

Connections that were selected in both cohorts when using the negative FIQ model primarily involved cerebellar vermis lobules. Previous research using the CPM model also suggests an importance of these brain connections with regard to FIQ (Finn et al. 2015). For future studies, it would be interesting to assess the small-world properties of the cerebellum concerning ASD FIQ compared to NT subjects. Another difference is the absence of cingulate ROIs for the positive ASD VIQ model compared to the NT VIQ model. These results agree with (Kilroy et al. 2011) as the cingulate receives multiple inputs and is responsible for effective emotional processing in NT subjects. For example, to respond effectively within a social context one would have to emotionally process the context of words within a conversation before giving a response. Conversely, this suggests a defect in autism as they are unable to correctly emotionally process information.

Overall, our models indicate altered connectivity differences for both VIQ and FIQ in ASD. As neural pruning occurs until the age of 25, this affects the nodal and global organization of networks. As our subjects were of adolescent age, neural pruning can affect brain functional connectivity and neural network organization (Hearne et al. 2016).

### Limitations

A limitation of our work is that we only considered low-order brain connectivity (i.e., between two ROIs), which might not well capture the breadth and complexity of neural activity in the brain. In fact, the brain wiring is a complex system that does not solely involve changes between pairs of ROIs but between sets of ROIs. Hence, using a multi-order brain network representation (Zhao et al. 2018; Lisowska and Rekik 2018a; Soussia and Rekik 2018) might enable a more comprehensive analysis of the neural basis of intelligence. Since the brain connectome can be derived from several MR imaging modalities (e.g., diffusion tensor imaging, T1-weighted, fMRI), one can integrate other network types such as structural connectomes (Cammoun et al. 2012) and morphological brain networks (Soussia and Rekik 2017; Lisowska et al. 2017; Mahjoub et al. 2018; Zhou et al. 2018) in a unified framework to examine brain-intelligence in a more holistic manner. Comparatively, we used whole-brain connectivity measures for the identification of ROIs in relation to IQ scores for both cohorts. Whole-brain connectivity is limited as it does not comprise of higher-order association cortices and exhibits higher inter-subject variance (Finn et al. 2015). We also note that the p-value threshold for identifying the most significant connections was fixed empirically. However, one can use nested cross-validation techniques to automatically tune in this statistical parameter for each training set in each run. Another limitation of our study is that we model the relationship between significant features and intelligence scores as a linear one. However, it may be important to model non-linear components of this relationship, in which case more advanced non-linear regression and machine learning techniques including robust nonlinear discriminant analysis and kernel support vector regressor combined with advanced feature selection methods can be leveraged (Huang et al. 2016; Lisowska and Rekik 2018b). If the goal is to maximize the prediction accuracy, one may consider using one of the multivariate regression methods, comparing the results with CPM, and selecting the method that gives the best prediction. Lastly, in this study, we have only focused on evaluating VIQ and FIQ scores. However, one can investigate other scores that quantify brain intelligence. For instance, there is evidence for a higher PIQ (performance IQ) – VIQ profile particularly in ASD subjects with higher levels of social impairment (Charman et al. 2010). If ASD subjects are socially avoidant, then one can assume standard neural pathways involved in VIQ would not be used as effectively as in NT subjects (Charman et al. 2010).

We intend to explore these directions in our future work as well as validate our intelligence-related discoveries using larger datasets.

## Conclusion

In this paper, we proposed prediction of intelligence scores from brain functional connectomes using a CPM protocol (Shen et al. 2017). More importantly, we identified positive and negative brain connectivities correlated with FIQ and VIQ intelligence scores in NT and ASD subjects. Our FIQ models suggests increased frontal and parietal connections in ASD. The negative model includes frequent cerebellar vermis connections for ASD and NT. Cerebellar connections have been postulated to be important for intelligence (Finn et al. 2015; Bullmore and Sporns 2009; Pezoulas et al. 2017). The VIQ model reports increased cingulate activity for the positive NT model. Rather than focusing on underconnectivity or overconnectivity of particular regions, our results suggest altered connectivity of ASD, similarly (Mostofsky and Ewen 2011; Kikuchi et al. 2013). In the future, it would be advisable to assess network associations to provide a clearer understanding of ASD connectivity. For example, investigating positive and negative features in association to network identification (e.g. fronto-parietal networks) for ASD, similar to Finn et. al (2015).

## Footnotes

1 https://github.com/preprocessed-connectomes-project/abide
